# *Corynebacterium tuberculostearicum*, a human skin colonizer, induces the canonical nuclear factor‐κB inflammatory signaling pathway in human skin cells

**DOI:** 10.1002/iid3.284

**Published:** 2020-01-07

**Authors:** Mohammed O. Altonsy, Habib A. Kurwa, Gilles J. Lauzon, Matthias Amrein, Anthony N. Gerber, Wagdi Almishri, Paule Régine Mydlarski

**Affiliations:** ^1^ Division of Dermatology, Department of Medicine University of Calgary Calgary Canada; ^2^ Department of Zoology, Faculty of Science Sohag University Sohag Egypt; ^3^ Department of Cell Biology and Anatomy University of Calgary Calgary Canada; ^4^ Department of Medicine National Jewish Health Denver Colorado; ^5^ Department of Medicine University of Colorado Denver Colorado; ^6^ Division of Gastroenterology, Department of Medicine University of Calgary Calgary Canada

**Keywords:** *Corynebacterium tuberculostearicum*, cutaneous squamous carcinoma cells, IKK, inflammation, keratinocytes, NF‐κB

## Abstract

**Introduction:**

*Corynebacterium tuberculostearicum* (*C. t*.) is a ubiquitous bacterium that colonizes human skin. In contrast to other members of the genus *Corynebacterium*, such as toxigenic *Corynebacterium diphtheriae* or the opportunistic pathogen *Corynebacterium jeikeium*, several studies suggest that *C. t*. may play a role in skin health and disease. However, the mechanisms underlying these effects remain poorly understood.

**Methods:**

To investigate whether *C. t*. induces inflammatory pathways in primary human epidermal keratinocytes (HEKs) and human cutaneous squamous carcinoma cells (SCCs), cell culture, reverse transcription‐polymerase chain reaction (PCR), enzyme‐linked immunosorbent assay, immunofluorescence microscopy, Western blot, chromatin immunoprecipitation‐PCR, small interfering RNA knockdown and luciferase reporter expression system were used.

**Results:**

Herein, we demonstrate that *C. t*. upregulates the messenger RNA (mRNA) and protein levels of inflammatory mediators in two human skin cell lines, HEKs and SCCs. We further show activation of the canonical nuclear factor‐κB (NF‐κB) pathway in response to *C. t*. infection, including phosphorylation of the inhibitor of κB (IκB), the nuclear translocation of NF‐κB subunit (NF‐κB‐P_65_) and the recruitment of NF‐κB‐P_65_ and RNA polymerase to the NF‐κB response elements at the promoter region of the inflammatory genes. Lastly, the data confirm that *C. t*.‐induced tumor necrosis factor mRNA expression in HEKs is toll‐like receptor 2 (TLR_2_) dependent.

**Conclusion:**

Our results offer a mechanistic model for *C. t*.‐induced inflammation in human keratinocytes via TLR_2_ and activation of IκB kinase and downstream signaling through the canonical NF‐κB pathway. Relevance to chronic inflammatory diseases of the skin and cutaneous oncology is discussed.

## Graphical abstract

*Corynebacterium tuberculostearicum* (*C. t*.) may play a role in skin health and disease. *C. t*. upregulates the messenger RNA (mRNA) and protein levels of inflammatory mediators in the human skin cell via the activation of the canonical nuclear factor‐κB pathway.

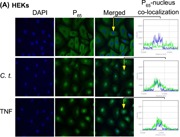

## 1 INTRODUCTION

Human skin and its constituent cells, including keratinocytes, provide the first line of defense at the interface with the environment, including against microbial pathogens.^1^ In healthy individuals, a symbiotic or mutualistic relationship exists between the host and its microbial flora. Dysbiosis refers to a disequilibrium of the microbial community with resultant effects on skin health and disease.^2^, ^3^, ^4^ Inflammatory skin diseases, such as atopic dermatitis, psoriasis, and rosacea, are postulated to be caused, at least in part, by an alteration of the normal but intricate equilibrium dictated by the environment, host genetics/immunity and the skin microbiota.^5^, ^6^, ^7^, ^8^, ^9^ For example, colonization of the skin by *Propionibacterium acnes* is causally related to facial acne.^10^, ^11^ In turn, the inflammatory and metabolic status of the host regulate the functional impact of microbes.^12^ Furthermore, host genetic factors are determinants of a skin microbiota predisposing to skin health or pathology; for example, the skin of patients with atopic dermatitis, a genetic disease related etiologically to mutations of the filaggrin (FLG) gene, demonstrates increased colonization with *Staphylococcus aureus* and *Staphylococcus epidermidis* (*S. e*.).^13^, ^14^

In healthy skin, commensals are unlikely to cause skin inflammation in the absence of a compromised epidermal barrier.^15^ In the context of a compromised barrier, keratinocytes are effective initiators of inflammation through the production of cytokines, chemokines, and adhesion molecules.^16^, ^17^ Furthermore, an exaggerated or skewed inflammatory response is likely crucial for the development of inflammatory skin diseases, as demonstrated for atopic dermatitis, psoriasis, alopecia areata, and systemic lupus erythematosus.^18^, ^19^, ^20^ Thus, understanding the mechanisms whereby skin microbiota impacts the keratinocytic regulation of inflammatory mediators and the induction of adhesion molecules would offer novel drug strategies for inflammatory skin diseases.

Bacteria of the genus *Corynebacterium* account for 30% of the total bacterial inhabitants of human skin.^3^ Some *Corynebacterium* species are opportunistic pathogens and coexist among healthy skin flora, for example, *Corynebacterium jeikeium* (*C. j*.),^21^ while other species, such as toxigenic *C. diphtheriae*, are classified as serious and potentially life‐threatening pathogens.^22^, ^23^ The species *C. tuberculostearicum* (*C. t*.), characterized by C. Feurer et al in 2004^24^ is a major component of the bacterial species that colonize a variety of skin environments, including dry, moist, and sebaceous regions.^25^ Several studies have associated *C. t*. with disease states, including inflammatory breast disease, pancreatic panniculitis, chronic rhinosinusitis, and surgical site infections.^26^, ^27^, ^28^, ^29^ However, the mechanisms by which *C. t*. plays a causative role in skin inflammation, remains unclear.

Herein, we applied the techniques of reverse transcription‐polymerase chain reaction (RT‐PCR), enzyme‐linked immunosorbent assay (ELISA), immunofluorescence microscopy, Western blot, chromatin immunoprecipitation‐PCR (ChIP‐PCR), small interfering RNA (siRNA) knockdown and luciferase reporter expression system to investigate whether *C. t*. induces inflammatory pathways in primary human epidermal keratinocytes (HEKs) and human cutaneous squamous carcinoma cells (SCCs). Our results provide conclusive evidence that *C. t*. activates the canonical nuclear factor‐κB (NF‐κB) pathway via toll‐like receptor 2 (TLR_2_), an effect regulated by the activation of IκB kinase (IKK). In parallel, we investigated the effects of *C. j*. and the commensal, but occasionally pathogenic, *S. e*. bacteria on the messenger RNA (mRNA) expression of the corresponding inflammatory genes in the same cell lines.

## 2 METHODS

### 2.1 Cell culture and reagents

HEKs cells (catalog no. C‐005‐5C; Gibco) were cultured in EpiLife growth medium (catalog no. MEPI500CA; Gibco) supplemented with human keratinocyte growth supplement (catalog no. S‐001‐K; Gibco). Cells were seeded on collagen type IV (catalog no. C5533; Sigma‐Aldrich) precoated plates. Human SCC cells (A431; ATCC, VA) were cultured in Dulbecco's modified Eagle's medium (catalog no.11995‐065; Gibco) supplemented with 10% fetal bovine serum (catalog no. 098105; Multicel). PS‐1145 dihydrochloride (catalog no. P6624; Sigma‐Aldrich) was dissolved in dimethyl sulfoxide (DMSO; catalog no. D5879; Sigma‐Aldrich) and diluted at least 1000‐fold in culture medium before treatment. Tumor necrosis factor (TNF; catalog no.210‐TA‐020; R&D Systems) was dissolved in phosphate‐buffered saline (PBS) containing 0.1% bovine serum albumin (BSA; catalog no. A3095; Sigma‐Aldrich). FSL1 (Pam2CGDPKHPKSF, catalog no. tlr‐fsl; InvivoGen).

### 2.2 Bacterial cultures and infection

*C. t*. (catalog no. 35693; ATCC) and *C. j*. (catalog no. 43734; ATCC) were cultured as described by Hinic et. al.^30^ Briefly, bacteria were cultured at 37°C for 24 hours on tryptic soy agar plates (catalog no. 236950; DIFCO) supplemented with 5% defibrinated sheep blood (catalog no. 610‐100; Quad Five, MT) and 1% Tween‐80 (catalog no. P1754; Sigma‐Aldrich) to achieve clearly visible colonies. Single colonies were used to inoculate tubes containing 10 mL each of Bacto tryptic soy broth (catalog no. 211825; BD Biosciences, CA) supplemented with 0.2% Tween‐80 and incubated at 37°C with continuous shaking until the desired optical density (OD) was reached (Figure S1). *S. e*. (catalog no. 14990; ATCC) was grown on No. 3 agar plates (catalog no. BD 213000; ATCC), and single colonies were inoculated to tubes containing 10 mL each of No. 3 broth (catalog no. BD 234000; ATCC). All bacteria were collected at late exponential growth phase (OD = 0.8 for *C. t*.; 1.5 for *C. j*.; 1.2 for *S. e*. as measured at 660 nm using SPECTRAmax PLUS384 spectrophotometer) and used to infect HEKs or SCC cells at a multiplicity of infection (5:1, bacteria:cells).

### 2.3 RNA purification, complementary DNA synthesis, and RT‐PCR

Total RNA was purified from HEKs and SCC cells using NucleoSpin RNA purification kits (catalog no. 740955‐ 250; D‐MARK Biosciences) following the manufacturer's instructions. Five‐hundred nanograms were used for complementary DNA (cDNA) synthesis using qScript cDNA Synthesis kit (catalog no. CA101414‐098; Quanta Biosciences). RT‐PCR was performed using Fast SYBR Green master mix (catalog no. 4385618; Life Technologies) and StepOne Plus thermal cycler machine (Applied Biosystems, Foster City, CA). Amplification conditions were 95°C for 20 seconds followed by forty cycles of 95°C for 3 seconds and 60°C for 30 seconds. Primer sequences are listed in Table S1.

### 2.4 Enzyme‐linked immunosorbent assay

Following each treatment, culture media were collected, centrifuged at 1000*g* for 5 minutes and the released cytokines were quantified using R&D Systems kits for interleukin 6 (IL6) (catalog no. Dy206), IL8 (catalog no. DY208), CSF3 (catalog no. DY214), IL1β (catalog no. DY201), CXCL10 (catalog no. DY266), and ICAM1 (catalog no. DY720), following the manufacturer's instructions. ELISA plates were read on SPECTRAmax PLUS384 Microplate spectrophotometer set to 450 and 540 nm; for wavelength correction, readings at 540 nm were subtracted from the readings at 450 nm. The concentration of cytokines was extrapolated using the third‐order polynomial (cubic) equation generated using the absorbance and concentration values of each cytokine's standard (provided with the kit). Paired *t* tests, performed on the GraphPad Prism 6 statistics software, were used to calculate the significance between cytokine concentrations of *C. t.‐* and TNF‐treated cells, relative to control cells.

### 2.5 Immunofluorescent microscopy

Cells were grown as a monolayer in an eight‐well chamber slide (catalog no. 177402; Lab‐Tek NALGE NUNC INTERNATIONAL). After the indicated treatments, cells were fixed in ice‐cold methanol (catalog no. A412; Fisher Chemicals) for 10 minutes at −20°C. Cells were then blocked for 1 hour at room temperature in 1% BSA (catalog no. a‐4503; Sigma‐Aldrich) dissolved in PBS containing 0.01% Tween 20 (catalog no. P5927; Sigma‐Aldrich). Cells were subsequently incubated overnight at 4°C with antibodies against phosphorylated IκBα (mouse monoclonal antibody [catalog no. 9246; Cell Signaling]), NF‐κB‐P_65_ (mouse monoclonal antibody [catalog no. SC‐293072; Santa Cruz Biotechnology]) or TLR_2_ (rabbit monoclonal antibody [catalog no. 12276; Cell Signaling]) in PBS‐Tween‐BSA at the manufacturer‐recommended dilutions. After this incubation, cells were washed three times (5 minutes each) in PBS and incubated with Alexa Fluor 488 goat anti‐mouse secondary antibody (catalog no. A11029; Invitrogen) diluted in PBS‐Tween‐BSA (1:500) for 1 hour at room temperature, followed by three washes (5 minutes each) in PBS. For nuclear counterstain, cells were incubated for 5 minutes at room temperature in PBS containing 4′,6‐diamidino‐2‐phenylindole (catalog no. d21490; Molecular Probes) at a concentration of 300 nM and washed three times (5 minutes each) in PBS. Immunoprobed cells were mounted using prolong gold antifade reagent (catalog no. p36930; Invitrogen) and visualized with confocal microscopy (Zeiss, Oberkochen, Germany) using ZEN 2012 software. Mean fluorescence intensity was calculated using the mean gray value analysis tool in the ImageJ software.

### 2.6 Subcellular fractionation

Subcellular fractionation was performed as previously described,^31^ with the following modifications: HEKs or SCC cells were grown in six‐well plates and, after the indicated treatments, were washed twice in cold PBS, scraped and transferred to 1.5 mL tubes. Cells were collected by centrifugation at 250*g* for 5 minutes at 4°C and resuspended in 250 μL of subcellular fractionation buffer (sucrose, 250 mM; 4‐(2‐hydroxyethyl)‐1‐piperazineethanesulfonic acid, 20 mM, pH 7.4; KCL, 10 mM; MgCl_2_, 1.5 mM; ethylenediaminetetraacetic acid, 1 mM; egtazic acid, 1 mM; dithiothreitol, 1 mM; 100 × Halt protease inhibitor cocktail (1%, catalog no. 1861279; Thermo Fisher Scientific), and incubated on a roller for 30 minutes at 4°C. Cell lysates were centrifuged at 720*g* for 5 minutes at 4°C, and the supernatant (cytoplasmic fraction) was collected in a fresh tube. The pellet (nuclei) was washed with 250 μL of the subcellular fractionation buffer and suspended in 100 μL of nuclear lysis buffer (Tris‐HCl, 1M [pH 8]; NaCl, 1M; NP‐40, 1%; sodium deoxycholate, 0.5%; sodium dodecyl sulfate [SDS], 0.1%; glycerol, 10%; 100X Halt protease inhibitor cocktail, 1%). The nuclear suspension was sonicated on ice with a Diagenode Bioruptor at high power in 30‐seconds bursts separated by 30‐seconds resting for a total of 5 minutes, yielding the nuclear fraction.

### 2.7 Electrophoresis and Western blot analysis

Cellular lysates were prepared in radioimmunoprecipitation assay buffer (sodium chloride, 150 mM; NP‐40, 1%; sodium deoxycholate, 0.5%; Tris, 50 mM [pH 8]; SDS, 1%; 100X Halt protease inhibitor cocktail, 1%), sonicated with Branson 2510 sonicator for 30 minutes at 4°C and the total protein concentration determined using Bio‐Rad protein assay (catalog no. 500‐0006; Bio‐Rad). Protein samples were denatured by the addition of 2X Laemmli buffer (SDS, 4%; β‐mercaptoethanol, 10%; glycerol, 20%; bromophenol blue, 0.004%; Tris‐HCl, 0.125M), 1:1 (vol/vol) and boiled at 95°C for 5 minutes. Fifty micrograms of protein were separated electrophoretically on a 10% SDS‐polyacrylamide gel. BLUelf prestained protein ladder (catalog no. PM008‐0500; FroggaBio) was used as a molecular weight marker. Proteins were transferred from resolved gels to nitrocellulose membranes (catalog no. rpn203d; GE Healthcare). Membranes were blocked using 5% nonfat dry milk (catalog no. 1706404XTU; Bio‐Rad) in Tris‐buffered saline‐Tween 20 (Tris‐HCI, 20 mM; NaCl, 500 mM; Tween 20, 0.05% [pH 7.5]) for 2 hours on a rocker platform at room temperature and probed with the following: phosphorylated IκBα mouse monoclonal antibody (catalog no. 9246; Cell Signaling), NF‐κB‐P_65_ mouse monoclonal antibody (catalog no. SC‐293072; Santa Cruz Biotechnology), rabbit monoclonal anti‐human TLR_2_ (catalog no. 12276; Cell Signaling), mouse anti‐human glyceraldehyde 3‐phosphate dehydrogenase (GAPDH; catalog no. 4699‐9555; Biogenesis) or cyclic AMP‐responsive element‐binding protein (CREB) rabbit monoclonal antibody (catalog no. 9197; Cell Signaling). Corresponding secondary antibodies were peroxidase‐conjugated goat anti‐rabbit immunoglobulin G (IgG) (catalog no. 111‐035‐003; Jackson ImmunoResearch) or peroxidase‐affinipure goat anti‐mouse IgG (catalog no. 115‐035‐003; Jackson ImmunoResearch). Immune complexes were visualized using ECL TM prime WB reagents (catalog no. rpn2232sk; GE Healthcare) and densitometric analysis was carried out using the TotalLab software (Nonlinear Dynamics).

### 2.8 Chromatin immunoprecipitation assay

HEKs or SCC cells were grown in complete medium in 100‐mm cell culture plates to 100% confluency and treated with *C. t*. bacteria at a ratio of 5:1 (bacteria:cells) or TNF (20 ng/mL) for 2 hours. Protein‐DNA cross‐linking was performed by the addition of 16% methanol‐free formaldehyde (catalog no. PI28906; Thermo Fisher Scientific) directly to the culture medium to a final concentration of 1%, and ChIP was performed as previously described.^32^ Purified DNA was analyzed by PCR. The quantitative (q)PCR‐ChIP primer sequences are listed in the Tables S2 and S3. Relative occupancy was calculated on a log_2_ scale based on comparison with the geometric mean of cycle threshold values (*C*_t_) for two negative control regions, as described.^33^ Antibodies used for ChIP were NF‐κB‐P_65_ mouse monoclonal antibody (catalog no. SC‐293072; Santa Cruz Biotechnology) and RNA polymerase II (RNA Pol‐II) antibody (catalog no. 920102; BioLegend). NF‐κB putative binding loci were identified using the TFBIND online tool (http://tfbind.hgc.jp/) and UCSC genome browser database.

### 2.9 Transfections and luciferase assays

HEKs and SCC cells were plated in 250 μL of antibiotic‐free complete growth medium in 48‐well plates at a density of 4 × 10^4^ cells/well and incubated overnight before plasmid transfection. A complex of 1 μL Lipofectamine 2000 (catalog no. 11668‐027; Invitrogen) and 400 ng DNA of pTNF3′NF‐κB plasmid^34^ or PGL3 promoter empty vector (E.V., catalog no. E1761; Promega) diluted in 50 μL Opti‐MEM (catalog no. 31985; Gibco) was added to each well and incubated for 48 hours. Transfected cells were treated for 6 hours with *C. t*. bacteria or TNF as indicated and luciferase activity was then assayed as previously described.^33^ For siRNA transfection, HEKs cells were plated onto 12‐well plates at a density of 1.5 × 10^5^ for 24 hours and transfected with 25 nM of ON‐TARGETplus human TLR2 (7097) siRNA‐SMARTpool (catalog no. L‐005020‐01; Dharmacon) or FlexiTube Lamin A/C nontargeting siRNA, ctrl‐siRNA (catalog no. SI03650332; Qiagen) using Lipofectamine RNAiMAX transfection reagents (catalog no. 13778075; Thermo Fisher Scientific) following the manufacturer's protocol.

### 2.10 Statistical analysis

Data were obtained from *N* independent biological experiments and are depicted as plots of the mean of individual values with SD error bars, or box‐and‐whisker plots showing the median, the 25th, and the 75th quartiles, as well as the minimum to maximum values. Technical triplicates were performed for qPCR, ELISA, Western blot densitometric analysis, ChIP‐qPCR, and luciferase data unless otherwise indicated. The GraphPad Prism 6 software was used for statistical analyses. The Student *t* test was used to determine the significant difference between cells treated with *C. t., C. j., S. e*., or TNF and nonstimulated (NS) cells. One‐way analysis of variance was performed with Tukey's correction for multiple comparisons. Heatmaps were generated using the Cluster 3.0 software.^35^ The values of log_2_‐fold change in the mRNA expression levels, referred from RT‐PCR data, were filtered by removing all genes with standard deviations for observed values of less than one, centered based on the values of the mean of mRNA expression in different treatments and clustered based on the similarity metric‐uncentered correlation among the tested genes.

## 3 RESULTS

### 3.1 *C. t*. upregulates the mRNA and protein levels of inflammatory mediators in HEKs and SCC cells

**Figure 1:**
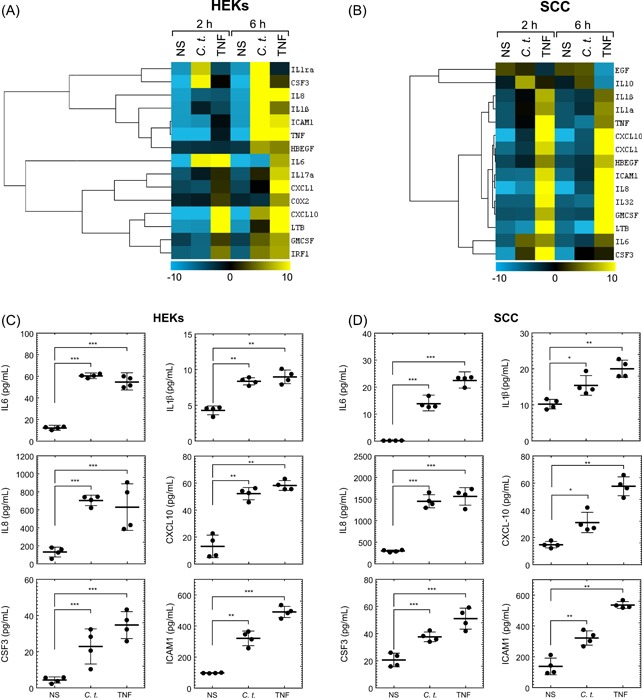
*Corynebacterium tuberculostearicum* (*C. t*.) upregulates mRNA and protein expression of inflammatory genes in HEKs and SCC cells. Heatmap and cluster analysis were generated using the Cluster 3.0 software as indicated in the methods section. The normalized log_2_ fold change in mRNA expression of 15 inflammatory genes is shown in response to infection with *C. t*. or treatment with TNF in HEKs (A) or SCC cells (B). The color bar demonstrates the log_2_ fold change where yellow and blue denote high and low mRNA expression, respectively, *N* = 4. ELISA analysis of the released inflammatory proteins measured in the culture medium is shown for HEKs (C) and SCC cells (D) treated with *C. t*. or TNF for 8 hours, *N* = 4, and expressed as concentrations pg/mL depicted as individual values scatter plots with SD bars using the GraphPad Prism 6 software. The Student *t* test was used to determine the significant difference between the two treatment groups. ELISA, enzyme‐linked immunosorbent assay; HEK, human epidermal keratinocyte; IL, interleukin; mRNA, messenger RNA; NS, nonstimulated; SCC, squamous carcinoma cell; TNF, tumor necrosis factor. *P < .05; **P < .01; ***P < .001

Keratinocytes contribute to the barrier functions of the epidermis through the formation of tight junctions and the stratum corneum and mediate inflammation through the secretion of cytokines, chemokines, and antibacterial peptides, and the expression of cellular adhesion molecules.^17^, ^36^, ^37^, ^38^ Accordingly, we evaluated the inflammatory response of primary HEKs and human SCC cells to in vitro infection by *C. t*. bacteria. Cells treated with TNF, a typical inflammatory cytokine, served as a positive control for the induction of inflammatory genes. The mRNA of 28 genes involved in the inflammatory response to bacterial infections were quantified using qRT‐PCR. Data analysis shows significant upregulation by *C. t*. of a group of proinflammatory genes in HEKs and SCC cells (Figure 1A,B), although the effects of *C. t*. on the expression of specific mRNAs varied between the two cell types. Infection with *C. t*. upregulated genes for IL1ra, IL17a, and IRF1 in HEKs cells only, while the corresponding treatment upregulated IL10 in SCC cells exclusively. The remaining upregulated mRNA species demonstrated increases in both HEKs and SCC cells (Figure 1A,B). The mRNA expression profile of the tested genes was time‐dependent following *C. t*. infection. In HEKs, genes for IL1ra, CSF3, IL1β, IL6, and IL17a were rapidly induced within 2 hours of *C. t*. infection, whereas the corresponding early responding genes in SCC cells were IL10, IL1β, IL1α, TNF, CXCL10, CXCL1, HBEGF, IL6, and CSF3. Enhanced transcription of the remaining upregulated genes in HEKs and SCC cells was detected 6 hours after infection (Figure 1A,B). The release of select inflammatory proteins (IL6, IL8, CSF3, IL1β, CXCL10, and ICAM1) into the culture medium of HEKs and SCC cells following infection with *C. t*. or treatment with TNF for 8 hours was quantified using ELISA (Figure 1C,D). The culture medium concentrations of the six cytokines for both cell types were significantly increased following the treatments with *C. t*. or TNF. Together with the mRNA data above, this provides confirmation that *C. t*. elicits an inflammatory response, although qualitatively and quantitatively different in HEKs and SCC cells.

### 3.2 Differential effects of *C. t., C. j*., and *S. e*. on mRNA expression in HEKs and SCC cells

**Figure 2:**
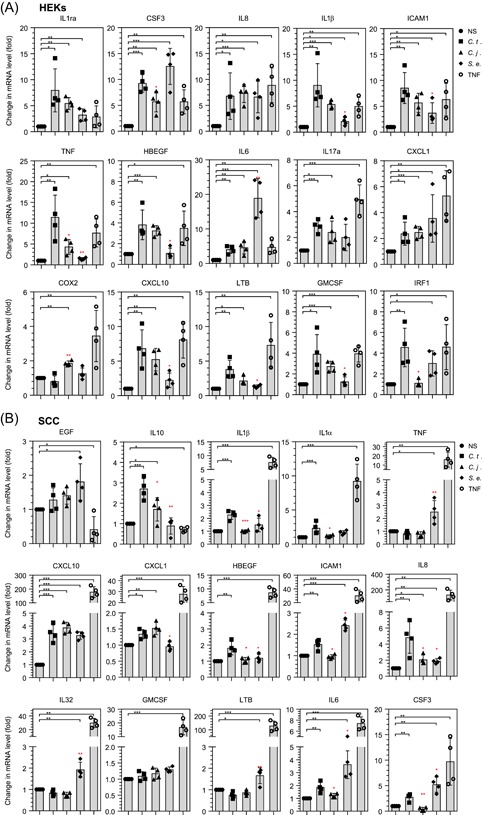
Differential effects of *Corynebacterium tuberculostearicum* (*C. t*.), *Corynebacterium jeikeium* (*C. j*.), and *Staphylococcus epidermidis* (*S. e*.) on the mRNA expression of inflammatory cytokines. HEKs (A) and SCC cells (B) were infected with *C. t., C. j*., or *S. e*., or treated with TNF for 6 hours, as described in the methods section. mRNA transcripts of upregulated genes for HEKs and SCC cells (see Figure 1) were quantified using RT‐PCR and normalized to the mRNA expression values of RPL19 gene from the respective samples. Data for *N* = 4 presented as bars and scatter plot of individual values representing fold‐change of mRNA levels, with SD error bars. Significance was obtained by comparing the expression of specific mRNAs in nonstimulated (NS) (black‐colored stars) or *C. t*.‐infected cells (red‐colored stars) using the Student *t* test. HEK, human epidermal keratinocyte; IL, interleukin; mRNA, messenger RNA; RT‐PCR, reverse transcription‐polymerase chain reaction; SCC, squamous carcinoma cell; TNF, tumor necrosis factor. *P < .05; **P < .01; ***P < .001

*C. j*. is an opportunistic human pathogen reported to induce inflammatory cytokines, such as IL17a.^12^, ^21^, ^39^ *S. e*. is a cutaneous commensal in humans, which can become an opportunistic pathogen, accounting for ∼22% of systemic infections in intensive care patients in the United States.^40^ We infected HEKs and SCC cells with *C. t., C. j*., or *S. e*. for 6 hours and quantified the mRNA expression of the genes that showed significant induction by treatment with *C. t*. or TNF in the experiments of Figure 1. The three bacterial strains affected the mRNA expression of the tested genes differently in HEKs and SCC cells (Figure 2). Significant differences between infection of HEKs with *C. t*. or *C. j*. (*P* ≤ .05) were identified for CSF3, TNF, and IRF1 genes. Their corresponding mRNA expression levels showed increments greater than fivefold in response to *C. t*. infection, but significantly less or no expression following treatment with *C. j*. (Figure 2A). Infections of HEKs cells with either *C. t*. or *C. j*. increased the mRNA transcripts of IL1ra, IL8, IL1β, ICAM1, HBEGF, IL6, IL17a, CXCL1, CXCL10, LTB, and GMCSF, compared with NS controls (*P* ≥ .05; Figure 2A). Conversely, COX2 mRNA levels were significantly higher in *C. j*.‐ relative to *C. t*.‐infected cells.

*S. e*.‐infected HEKs showed significant (*P* ≤ .05) induction of mRNA levels for IL1ra, CSF3, IL8, IL1β, ICAM1, IL6, CXCL1, and IRF1 (Figure 2A). However, the mRNA coding for IL1β and ICAM1 were significantly lower as compared to their corresponding mRNA expression levels in *C. t*.‐infected cells (Figure 2). Notably, the expression of mRNA for TNF, HBEGF, CXCL10, LTB, and GMCSF was not affected by *S. e*. infection, relative to NS controls. Interestingly, Figure 2A shows that *S. e*. infection of HEKs increased the mRNA expression of the IL6 gene very significantly (18.78‐fold; *P* ≤ .001), compared with *C. t*. infection (fourfold; *P* ≤ .01) or *C. j*. infection (4.56‐fold; *P* ≤ .01).

In SCC cells, the mRNA expression of IL10, IL1β, IL1α, HBEGF, ICAM1, IL8, IL6, and CSF3 was significantly higher in *C. t*.‐ as compared with *C. j*.‐infected cells (Figure 2B), whereas the mRNA expression for CXCL10 and CXCL1 showed essentially identical responses to *C. t*. or *C. j*. infection. In *S. e*.‐infected SCC cells, the mRNA levels of CXCL1 and IL8 genes were significantly lower than in *C. t*.‐infected cells, while genes encoding TNF, ICAM1, IL32, LTB, and CSF3 were significantly higher than those in *C. t*.‐infected cells (*P* ≤ .05). *S. e*. infection of SCC cells did not affect mRNA levels of IL10, IL1β, IL1α, HBEGF, and GMCSF, compared with NS cells. The mRNA expression of EGF, TNF, IL32, GMCSF, and LTB was not affected by the treatment of SCC cells with *C. t*. relative to control NS‐treated cells. These data collectively highlight the unique effects of the three bacterial strains on the mRNA expression of proinflammatory genes in HEKs and SCC cells.

### 3.3 *C. t*. infection induces IκB phosphorylation and NF‐κB‐P_65_ nuclear translocation

Inactive NF‐κB, the archetypal inflammatory transcription factor, resides in the cytoplasm bound to IκB protein. Microbial infection or exposure to proinflammatory cytokines, such as TNF, triggers the canonical NF‐κB pathway via the activation of IKK which phosphorylates IκB, thereby liberating the NF‐κB‐P_65_ subunit that translocates to the nucleus and binds to DNA at specific loci, known as NF‐κB‐elements. This enhances the transcription of target genes, including those encoding numerous inflammatory effector molecules.^41^, ^42^ Results presented above show the activation by infection with *C. t*. of the transcription of a group of inflammatory genes. We studied the potential contribution of the canonical NF‐κB pathway to the *C. t*.‐induced transcription of proinflammatory genes, including the phosphorylation of IκB and nuclear translocation of NF‐κB‐P_65_ subunit in HEKs and SCC cells.

**Figure 3:**
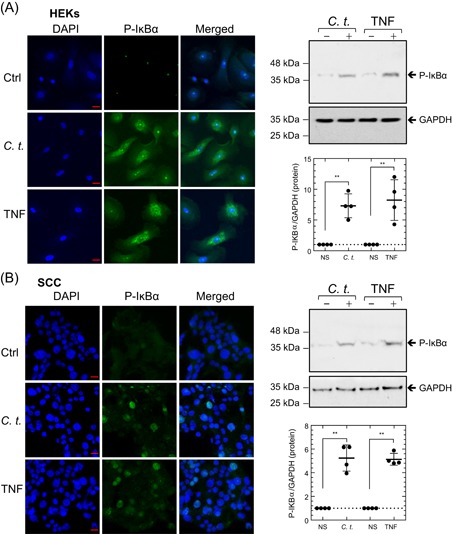
*Corynebacterium tuberculostearicum* (*C. t*.) infection rapidly induces IκB phosphorylation. Fluorescent micrographs and Western blot analysis show the dramatic increase of P‐IκB levels in HEKs (A) and SCC cells (B) 20 minutes after *C. t*. infection or treatment with TNF. Cells were immuno‐stained with an antibody specific for P‐IκB (green). DAPI (blue) was used as a nuclear DNA counterstain. Fluorescent images were acquired using the Zeiss laser scanning microscope and ZEN‐2012 software. Original magnification: ×630 (scale bar = 20 μm). Western blots (right upper panels) and densitometric quantification (right lower panels) of P‐IκB and GAPDH protein bands depicted as arbitrary scan units normalized to the values of protein band intensity of nonstimulated cells and plotted as individual values scatter plots with SD bars. Statistical significance was tested between the controls and *C. t‐*. or TNF‐treated samples using the Student *t* test. DAPI, 4′,6‐diamidino‐2‐phenylindole; GAPDH, glyceraldehyde 3‐phosphate dehydrogenase; HEK, human epidermal keratinocyte; P‐IκB, phosphorylation of IκB; SCC, squamous carcinoma cell; TNF, tumor necrosis factor. *P < .05; **P < .01; ***P < .001. Data, *N* = 4

We treated HEKs and SCC cells with *C. t*. or TNF for 20 minutes, based on prior kinetic studies, which showed that the phosphorylation of IκB takes place within 1 to 20 minutes of stimulus exposure.^43^ Phosphorylation of IκB (P‐IκB) was assessed by fluorescent microscopy and Western blot analysis, as described in Section 2. P‐IκB increased dramatically after *C. t*. infection or TNF treatment of HEKs (Figure 3A) and SCC cells (Figure 3B). Densitometric quantification of Western blot bands corresponding to P‐IκB demonstrated significant increases in HEKs cells infected with *C. t*. (7.31‐fold; *P* ≤ .0063) or treated with TNF (8.24‐fold; *P* ≤ .0054), relative to control cells (Figure 3A). Correspondingly, P‐IκB levels were significantly elevated in SCC cells infected with *C. t*. (5.24‐fold; *P* ≤ .0047) or treated with TNF (5.13‐fold; *P* ≤ .0212), as compared with control cells (Figure 3B).

**Figure 4:**
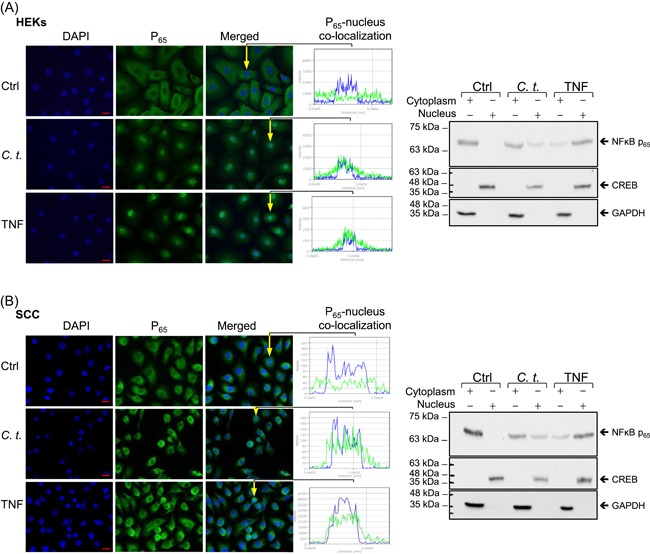
Infection with *Corynebacterium tuberculostearicum* (*C. t*.) induces nuclear translocation of NF‐κB‐P_65_. Fluorescent micrographs (left) show NF‐κB‐P_65_ (green) homogenous cytoplasmic distribution and essentially clear nuclei in control cells vs the nuclear‐localized NF‐κB‐P_65_ in cells treated for 1 hour with *C. t*. or TNF in HEKs (A) or SCC cells (B). DAPI (blue) was used as a nuclear marker and nucleus/NF‐κB‐P_65_ colocalization single‐cell profiles were generated using the laser scanning microscope and ZEN‐2012 software (original magnification: ×630; scale bar = 20 μm). Representative Western blots on SDS‐PAGE (right) of NF‐κB‐P_65_ derived from the cytoplasmic and nuclear fractions showed for HEKs (A) and SCC cells (B), after 1‐hour exposure to *C. t*. or TNF. Antibodies against CREB and GAPDH were used as markers for nuclear and cytoplasmic proteins, respectively. CREB, cyclic AMP‐responsive element‐binding protein; Ctrl, control; DAPI, 4′,6‐diamidino‐2‐phenylindole; GAPDH, glyceraldehyde 3‐phosphate dehydrogenase; HEK, human epidermal keratinocyte; NF‐κB, nuclear factor‐κB; SCC, squamous carcinoma cell; SDS‐PAGE, sodium dodecyl sulfate‐polyacrylamide gel electrophoresis; TNF, tumor necrosis factor

As the cellular localization of NF‐κB‐P_65_ between cytoplasmic and nuclear compartments fluctuates over short 1‐hour cycles,^44^, ^45^ we assessed NF‐κB‐P_65_ translocation 1‐hour after *C. t*. infection or TNF treatment. Fluorescence microscopic examination of control cells revealed the homogenous distribution of NF‐κB‐P_65_ in the cytoplasm and its absence in nuclei. In *C. t*.‐infected or TNF‐treated cells, NF‐κB‐P_65_ was primarily localized to the nucleus (Figure 4A,B). NF‐κB‐P_65_ translocation was further assessed using subcellular fractionation of cytoplasmic and nuclear proteins and Western blot analysis (Figure 4A,B). Antibodies against CREB and GAPDH were used as nuclear and cytoplasmic markers, respectively. In control cells, the nuclear content of NF‐κB‐P_65_ was undetectable by Western blot, whereas it was detected in the cytoplasmic and the nuclear compartments of *C. t*.‐ or TNF‐treated HEKs and SCC cells, confirming the translocation of NF‐κB‐P_65_ as a result of infecting HEKs and SCC cells with *C. t*.

### 3.4 IKK inhibitor (PS‐1145) attenuates *C. t*.‐induced expression of inflammatory genes in HEKs and SCC cells

**Figure 5:**
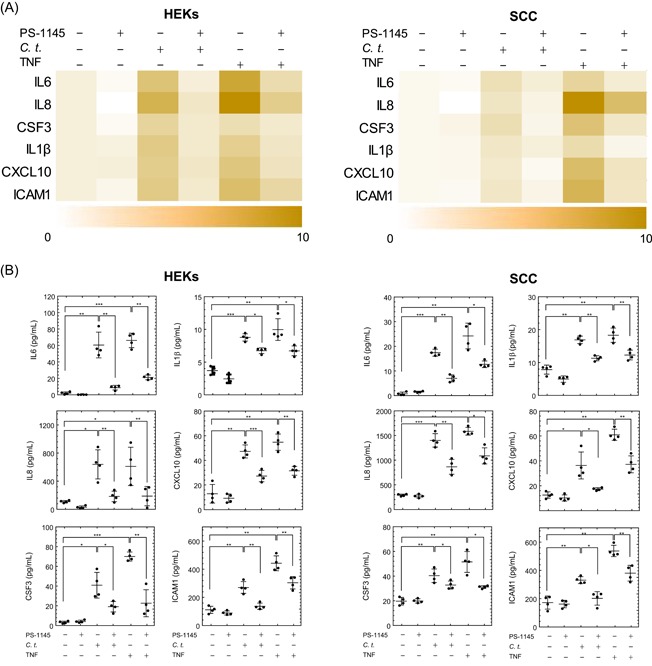
IKK inhibitor (PS‐1145) attenuates the mRNA and protein expression of the inflammatory genes induced by *Corynebacterium tuberculostearicum* (*C. t*.) infection. HEKs (panel A, left) and SCC cells (panel A, right) were treated with PS‐1145 (or DMSO) for 1 hour before *C. t*. or TNF exposure for 4 hours. Heatmaps depict the normalized log_2_ fold change of the mRNA expression derived from qRT‐PCR data for six inflammatory genes. The color bar demonstrates the log_2_ fold change where white and brown denote high and low mRNA expression, respectively (*N* = 4). ELISA analysis of the released inflammatory proteins measured in the culture medium of HEKs (panel B, left) or SCC cells (panel B, right) following 8 hours of *C. t*. or TNF treatment, with or without PS‐1145 pretreatment as indicated. Data for *N* = 4 expressed as concentrations (pg/mL) depicting individual values and SD bars displayed using the GraphPad Prism 6 software. The Student *t* test was used to determine the significant difference between the two treatment groups. DMSO, dimethyl sulfoxide; ELISA, enzyme‐linked immunosorbent assay; HEK, human epidermal keratinocyte; IKK, IκB kinase; IL, interleukin; mRNA, messenger RNA; qRT‐PCR, quantitative reverse transcription‐polymerase chain reaction; SCC, squamous carcinoma cell; TNF, tumor necrosis factor. *P < .05; **P < .01; ***P < .001

The canonical NF‐κB pathway involves IκB phosphorylation and NF‐κB translocation as downstream molecular events following IKK complex activation.^46^ To investigate the role of IKK in *C. t*.‐elicited transcription of inflammatory mediators, we treated HEKs and SCC cells with the IKK inhibitor PS‐1145 (10 μM) or DMSO vehicle control for 1 hour before incubating the cells with *C. t*. or TNF for an additional 4 hours. Cells were then harvested, RNA was isolated and six representative proinflammatory and NF‐κB‐dependent genes were quantitated by qRT‐PCR, including IL6, IL8, CSF3, IL1β, CXCL10, and ICAM1. These genes are known to be dysregulated in various human skin diseases,^16^, ^46^, ^47^, ^48^, ^49^, ^50^ and were shown above to be upregulated in response to *C. t*. or TNF in both cell types. As expected, *C. t*. as well as TNF significantly upregulated the mRNA expression of the above‐listed genes (Figure 5A). In contrast, the corresponding mRNA expression of these genes was dramatically decreased in PS‐1145‐treated cells, providing direct evidence that IKK is involved in the *C. t*.‐induced mRNA expression of these genes. The protein content of these six inflammatory mediators in the culture medium following 8 hours of culture with *C. t*. or TNF was quantified using ELISA (Figure 5B). Consistent with the mRNA data, treatment with *C. t*. or TNF significantly elevated the levels of the respective proteins in the culture medium. PS‐1145 significantly attenuated the inductive effect of *C. t*. and TNF on each of the released proteins. These data confirm that *C. t*. regulates IκB phosphorylation and NF‐κB‐P_65_ translocation to the nucleus and supports the claim that IKK is directly involved in the modulation of select proinflammatory genes by *C. t*. in both HEKs and SCC cells.

### 3.5 *C. t*. recruits NF‐κB‐P_65_ and RNA Poll‐II to NF‐κB response elements

**Figure 6:**
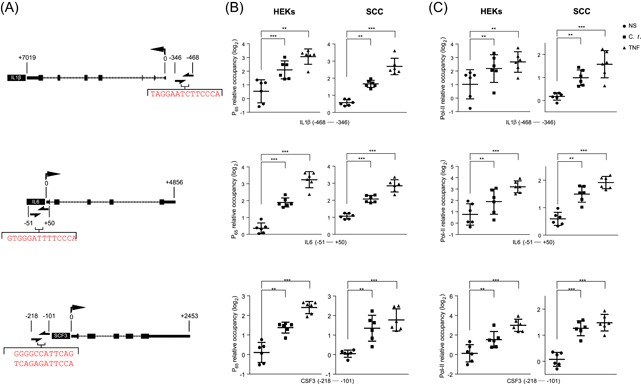
*Corynebacterium tuberculostearicum* (*C. t*.) increases the occupancy of NF‐κB‐P_65_ and RNA Pol‐II at the promoter regions of IL1β, IL6, and SCF3 genes. Panel A illustrates NF‐κB putative binding sequence(s) (nucleotides in red color) at the promoter regions of IL1β (top), IL6 (middle), and SCF3 (lower) genes identified in relation to the transcriptional start sites of the associated genes (half arrows indicate PCR primer loci). ChIP‐PCR analysis of the relative occupancy of NF‐κB‐P_65_ (B) or RNA Pol‐II (C) at the NF‐κB binding loci of the promoter regions of IL1β, IL6, and SCF3 are shown, calculated as a difference between *C*_t_ values for each target as compared with the geometric mean of *C*_t_ values of two control regions that are not occupied by either NF‐κB or RNA Pol‐II. Data for *N* = 6 expressed as log_2_ relative occupancy and depicted as individual values scatter with SD bar plots using the GraphPad Prism 6 software. The Student *t* test was used to determine the significant difference between the two treatment groups. ChIP‐PCR, chromatin immunoprecipitation‐PCR; HEK, human epidermal keratinocyte, IL, interleukin; NF‐κB, nuclear factor‐κB; PCR, polymerase chain reaction; RNA Pol‐II, RNA polymerase II; SCC, squamous carcinoma cell. *P < .05; **P < .01; ***P < .001

The above results addressing mRNA expression, cytokines/chemokine release, NFκB‐P_65_ nuclear translocation, and IκB phosphorylation collectively imply activation of the canonical NF‐κB pathway following infection with *C. t*. bacteria. Downstream signaling further involves the binding of NFκB‐P_65_ subunit and RNA Pol to DNA at the transcriptionally active loci.^51^, ^52^, ^53^ To confirm this linear process, ChIP‐PCR was applied to show the recruitment of NF‐κB‐P_65_ and RNA Pol‐II to the NF‐κB putative binding sequence at the promoter regions of IL1β, IL6, and CSF3 genes after 2 hours of exposure to *C. t*. or TNF (Figure 6). The occupancy of NF‐κB‐P_65_ and Pol‐II was calculated relative to the nonoccupied control regions as indicated in the methods section. Occupancy data are expressed on a log_2_ scale in Figure 6B,C. Data analyses indicate that in both cell types, infection with *C. t*. or treatment with TNF significantly increased the binding of NF‐κB‐P_65_ and Pol‐II to the promoter regions of the tested genes. Correlating the increased promoter region occupancy with the above mRNA data supports the contention that *C. t*. upregulates the expression of the studied inflammatory mediators through activation of the canonical NF‐κB pathway.

### 3.6 *C. t*. drives the transcription of pTNF3′NF‐κB (TNF luciferase reporter), and the luciferase activity is attenuated by PS‐1145

**Figure 7:**
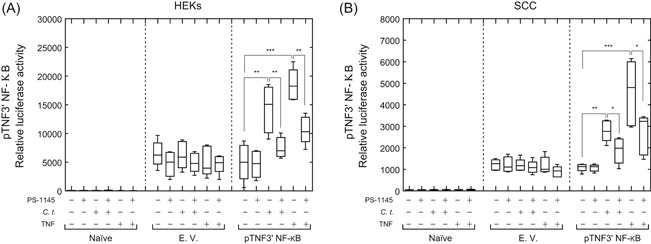
*Corynebacterium tuberculostearicum* (*C. t*.) induces the expression of pTNF3′NF‐κB luciferase construct in HEKs and SCC cells, and the luciferase activity is attenuated by PS‐1145. HEKs (A) or SCC cells (B), naïve or transfected with pGL3 promoter empty vector (E.V.) or with pTNF3′NF‐κB plasmid were treated for 1 hour with PS‐1145 (10 μM) or vehicle control (DMSO) before infection with *C. t*. or treatment with TNF. After 6 hours, luciferase activities were measured and collected data, *N* = 5, was analyzed with the GraphPad Prism 6 software. A two‐way analysis of variance was performed with Tukey's correction for multiple comparisons. The Student *t* test was used to determine the significant difference between two treatment groups, *P < .05; **P < .01; ***P < .001. Data are expressed as relative luciferase activity and depicted as box‐and‐whiskers plots showing the median, the 25th, and 75th quartiles, as well as a minimum to maximum values. DMSO, dimethyl sulfoxide; HEK, human epidermal keratinocyte; NF‐κB, nuclear factor‐κB; SCC, squamous carcinoma cell; TNF, tumor necrosis factor

To study the direct inducing effects of *C. t*. on the transcriptional activities of the proinflammatory mediators studied above, we utilized our previously cloned TNF reporter (pTNF3′NF‐κB),^34^ constructed by cloning the NF‐κB binding elements from the 3*′*‐untranslated region at the TNF gene to the region upstream of the luciferase gene in the pGL3 promoter vector. HEKs and SCC cells were transfected with the plasmid and treated with *C. t*. or TNF for 6 hours and the luciferase activities were measured as described in Section 2 (Figure 7A,B). *C. t*. infection increased the basal luciferase activity 2.85‐fold (*P* ≤ .032) and 2.55‐fold (*P* ≤ .001) in HEKs and SCC cells, respectively, indicating that *C. t*. is directly involved in the activation of an NF‐κB‐dependent transcription system. The effects of TNF were more pronounced and increased luciferase activity 3.68‐fold (*P* ≤ .0003) and 4.18‐fold (*P* ≤ .008) in HEKs and SCC cells, respectively. These data are consistent with our previous findings demonstrating the TNF‐induction of pTNF3′NF‐κB in bronchial epithelial cells.^34^ Treatment of HEKs with PS‐1145 for 1 hour before infection with *C. t*. or treatment with TNF reduced luciferase activity by 47.34% (*P* ≤ .018) and 42.44% (*P* ≤ .014) in *C. t*.‐infected and TNF‐treated cells, respectively. Correspondingly, in PS‐1145‐treated SCC cells, the luciferase activity was reduced 32.62% (*P* ≤ .012) and 41.64% (*P* ≤ .03) in the *C. t*.‐infected and TNF‐treated cells, respectively. Taken together, these data confirm that (a) *C. t*. stimulates the canonical NF‐κB pathway in HEKs and SCC cells, and (b) inhibiting the NF‐κB pathway activation using PS‐1145, an inhibitor of IKK, decreases the expression of *C. t*.‐induced inflammatory genes.

### 3.7 *C. t*. upregulates TLR_2_ mRNA and protein expression

**Figure 8:**
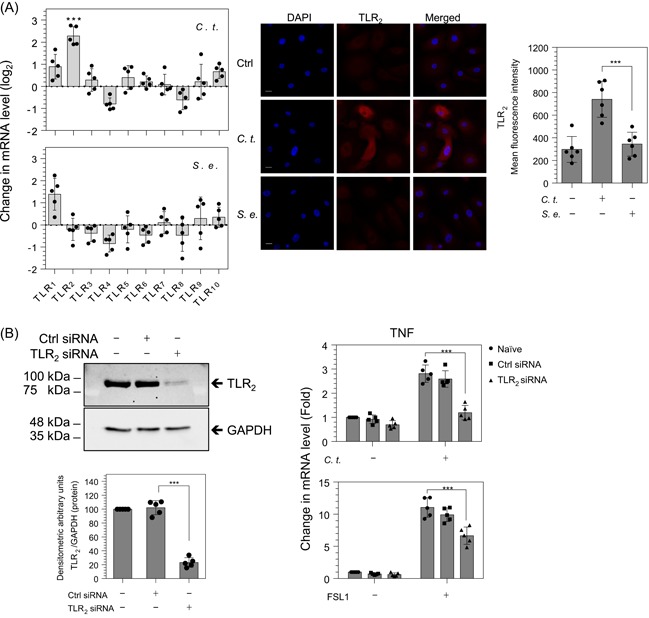
*Corynebacterium tuberculostearicum* (*C. t*.) increases TLR_2_ expression, and TLR_2_ siRNA reduces TNF mRNA induced by *C. t*. or FSL1 in HEKs. RT‐PCR analysis of mRNA expression of TLR _(1‐10)_ in HEKs infected with *C. t*. or *Staphylococcus epidermidis* (*S. e*.) for 6 hours (A, left panel). Fluorescent micrographs of control (Ctrl), *C. t*.‐ or *S. e*.‐infected HEKs were immunoprobed with TLR_2_ antibody (red). Original magnification: ×630 (scale bar = 20 μm), DAPI nuclear marker (blue) (A, middle panel). TLR_2_ expression was quantitated using the mean gray value analysis tool of the ImageJ software; MFI values are displayed as scatter plots with SD bars using the GraphPad Prism 6 software, *N* = 6. (A, right panel). Left panel (B) displays Western blot and densitometric quantification of TLR_2_ and GAPDH protein bands in HEKs treated with TLR_2_ siRNA or control siRNA for 48 hours under culture conditions. Right panel (B) displays RT‐PCR analysis of TNF mRNA expression in HEKs treated for 2 hours with or without *C. t*. and with or without FSL1, *N* = 5. The Student *t* test was used to determine the significant difference between the two treatment groups. DAPI, 4′,6‐diamidino‐2‐phenylindole; GAPDH, glyceraldehyde 3‐phosphate dehydrogenase; HEK, human epidermal keratinocyte; MFI, mean fluorescence intensity; mRNA, messenger RNA; RT‐PCR, reverse transcription‐polymerase chain reaction; siRNA, small interfering RNA; TLR_2_, toll‐like receptor 2; TNF, tumor necrosis factor. ***P < .001

To identify the cellular receptor involved in the activation of the NF‐κB pathway, we measured the mRNA expression of all known human TLR (1‐10) in HEKs in response to *C. t*. or *S. e*. infection. After 6 hours of treatment of HEKs with *C. t*., TLR_2_ mRNA was increased by 2.28 PCR cycles or 4.86‐fold (*P* ≤ .001), whereas mRNA for other TLR genes were not significantly increased (Figure 8A). Furthermore, mRNA of all TLR genes including TLR_2_ remained unaffected by exposure to *S. e*. (Figure 8A). At the protein level, immunocytochemistry and microscopic analysis confirmed that TLR_2_ protein was increased 2.37‐fold (*P* ≤ .001) after 24‐hour treatment of HEKs with *C. t*., relative to the basal expression in untreated HEKs. Meanwhile, infection with *S. e*. did not affect the protein content of TLR_2_ in HEKs.

### 3.8 TLR_2_ siRNA abrogates *C. t*.‐induced expression of TNF mRNA

To assess the role of TLR_2_ in *C. t*.‐elicited inflammation, we treated HEKs cells with nonspecific targeting (control) Ctrl‐ or TLR_2_‐siRNA for 48 hours before *C. t*.‐infection. Western blot analysis confirmed that TLR_2_ targeting siRNA significantly reduced TLR_2_ protein by 76.89% (*P* ≤ .001) (Figure 8B, left panel). TNF mRNA expression was measured in HEKs cells treated for 2 hours with *C. t*. or FSL1 (Pam2CGDPKHPKSF), a synthetic lipopeptide and a specific TLR_2/6_ agonist. As expected, *C. t*. increased the mRNA expression of TNF 2.81‐fold (*P* ≤ .001) and 2.59‐fold (*P* ≤ .001) in naïve and control siRNA‐treated cells, respectively, an effect that was abolished in TLR_2_ knockdown cells. Treatment with FSL1 increased levels of TNF mRNA 11.03‐fold (*P* ≤ .001) and 9.91‐fold (*P* ≤ .001) in naïve and control siRNA‐treated cells, respectively, and TLR_2_ knockdown significantly reduced the levels of TNF mRNA in FSL1‐treated cells to 6.65‐fold (*P* ≤ .001) (Figure 8B, right panel). These results suggest that TLR_2_ plays a role in *C. t*.‐induced inflammation in human keratinocytes. Moreover, the upregulation of TLR_2_ mRNA and protein in *C. t*.‐ but not in *S. e*.‐infected cells, is postulated to explain, at least partly, the relatively greater induction of NF‐κB inflammatory signaling pathway by *C. t*. as compared with *S. e*. (Figure 2A).

## 4 DISCUSSION

Through shotgun metagenomics, *C. t*. has been identified as an important colonizer of many skin microenvironments, whether dry, moist or sebaceous.^25^ *C. t*. disease associations have included inflammatory breast disease, pancreatic panniculitis, chronic rhinosinusitis, and surgical site infections.^26^, ^27^, ^28^, ^29^ Using 16S ribosomal RNA sequencing, our laboratory has also shown that *C. t*. is more commonly identified in keratinocyte carcinomas (ie, basal and squamous cell carcinomas) as compared with matched, perilesional skin controls (data presented at the Canadian Dermatology Association's 93rd Annual Conference). Taken together, these data suggest that *C. t*. may play an important role in skin health and disease.

As the major cellular component of the epidermis, keratinocytes have important barrier properties and are mediators of skin inflammation.^54^, ^55^ They produce various high‐molecular‐weight mucopolysaccharides, cytokines, chemokines, antimicrobial peptides, and cell adhesion molecules that recruit immune cells to sites of injury or infection.^16^, ^55^, ^56^ After a breach in the skin barrier, keratinocytes regulate an inflammatory response that mitigates microbial invasion.^57^ Unregulated and persistent cutaneous inflammation may lead to chronic inflammatory disease and, possibly, cancer.^58^, ^59^, ^60^, ^61^ Furthermore, data suggest that inflammatory responses to infection are linked to 15% to 20% of cancer deaths worldwide.^62^ We, therefore, asked the following question: does *C. t*. play a role in cutaneous inflammation and, if so, what are the underlying mechanisms?

The microbial infection has been shown to trigger the activation of TLR via the canonical NF‐κB pathway.^41^, ^42^, ^63^, ^64^ In this report, we demonstrate *C. t*., not only elicits inflammation but also upregulates the expression of TLR_2_, hence potentially amplifying and perpetuating the inflammatory response to TLR_2_ stimuli in human keratinocytes. These data collectively indicate that *C. t*. upregulated proinflammatory mRNAs and proteins involved in the NF‐κB pathway. Furthermore, in both HEKs and SCC cells, *C. t*., the opportunistic *C. j*. and the skin commensal *S. e*. showed differential mRNA expression. Our results support the notion that *C. t*. upregulates proinflammatory cytokines, chemokines, and cell adhesion molecules in keratinocytic cells, both primary cultures and SCC lines, through activation of the canonical NF‐κB pathway. In keeping with other bacterial pathogens, we confirmed the inflammatory role played by IKK.^65^, ^66^, ^67^, ^68^, ^69^ Using fluorescent microscopy and Western blot analysis, we demonstrated that phosphorylation of IκB is *C. t*.‐dependent. Furthermore, the inhibition of IKK by the specific inhibitor PS‐1145^70^ significantly reduced the mRNA and protein expression of proinflammatory molecules induced by *C. t*., indicating these effects are mediated, at least in major part, by IKK. P‐IκB, generated by IKK activation, is directed to proteasomal degradation, thereby allowing the translocation of NF‐κB‐P_65_ subunit to the nucleus and resultant transcription of target genes.^71^, ^72^ In agreement with this molecular pathway, our results confirm that *C. t*. promotes NF‐κB‐P_65_ nuclear translocation and subsequent attachment to the consensus binding sites of NF‐kB at the promoter region of IL1β, IL6, and CSF3 genes. Although transcription factor binding to cis‐ or trans‐acting DNA sequences is required to initiate transcription, it does not universally guarantee transcription; modification of the neighboring chromatin microenvironment is also needed to regulate the recruitment of RNA Pol and other cofactors to the transcriptionally active locus.^73^, ^74^, ^75^ Herein, we showed enhanced RNA Pol‐II occupancy at the promoter regions of IL1β, IL6, and CSF3 genes in the *C. t.‐* and TNF‐treated cells, confirming ongoing stimulus‐induced and active transcription.

As a reporter expression system recapitulates the activity of the DNA sequence from an actual gene, the pTNF3′NF‐κB luciferase reporter was used to provide quantitative measurements of transcription in response to *C. t*. stimulation. pTNF3′NF‐κB contains several NF‐κB binding sequences and is induced by TNF.^34^ In the present study, pTNF3′NF‐κB‐transfected HEKs and SCC cells exhibited significantly greater luciferase activity than that reported in nontransfected cells. Furthermore, the luciferase activities were significantly increased in keratinocyte‐derived cells treated with *C. t*. (which enhances cellular TNF; see Figure 1), and these effects were dramatically reduced in cells pretreated with PS‐1145, an inhibitor of IKK. Collectively, these results demonstrate that *C. t*. induced proinflammatory mediators in HEKs and SCC cells, in part by activation of the canonical NF‐κB pathway.

Although NF‐κB is a crucial regulator of host defenses, its dysregulation is associated with chronic inflammatory skin diseases.^76^, ^77^, ^78^ The *C. t*.‐mediated inflammatory effect in human keratinocytes is shown to be an NF‐κB‐dependent process, attenuated successfully by inhibition of IKK. We propose that *C. t*. could initiate and/or perpetuate chronic inflammatory diseases or malignancies of skin through subversion of the NF‐κB signaling pathway. The unique inductive effects of *C. t*., as compared with *C. j*. or *S. e*., on proinflammatory cytokines in keratinocyte‐derived cell lines support the potential for microbe‐specific predisposition to inflammatory skin diseases or malignancy. Future studies will investigate (a) the mechanistic impact of environmental, immunogenetic, and microbial determinants of the *C. t*. activation on inflammatory pathways in human skin; (b) the relevance of the specific inflammatory mediators induced by *C. t*. vs other microbes, and (c) the relevance of *C. t*. to keratinocyte carcinomas, including basal cell carcinoma and SCC.

## CONFLICT OF INTERESTS

The authors declare that there are no conflict of interests.

## AUTHOR CONTRIBUTIONS

MOA and PRM participated in research design; MOA, WA, and PRM conducted experiments and data acquisition; HAK, GJL, and MA contributed new reagent or analytical tool; MOA, HAK, GJL, MA, ANG, WA, and PRM performed data analysis; MOA, HAK, GJL, MA, ANG, WA, and PRM wrote or contributed to the drafting, writing, and critically reviewing of the manuscript; MOA and PRM gave final approval of the version to be published.

## ETHICS STATEMENT

The experiments reported in this article did not involve human nor animal samples. The model of the study was commercially available human cell lines (human epidermal keratinocytes and human cutaneous squamous carcinoma cells).

## Supporting information

Supporting information

## Data Availability

The data that support the findings of this study are available from the corresponding author upon reasonable request.
