# Supplementary material for: Corynebacterium tuberculostearicum, a human skin colonizer, induces the canonical nuclear factor‐κB inflammatory signaling pathway in human skin cells
Source: Immun Inflamm Dis. 2020 Jan 7;8(1):62–79. doi: 10.1002/iid3.284 (PMC7016847; doi:10.1002/iid3.284)
Supplement: Supplementary file 1 — Supporting information [file IID3-8-62-s001.docx]

**Supplemental Data**

**(A)**

**(B)**

**figure S1:** *A*. Growth curves of *C. tuberculostearicum*, *C. jeikeium*, and *S. epidermidis*. Bacteria were grown as indicated in the methods section and collected for plate counting at the late exponential/early stationary growth phase of each strain that corresponds to optical density (O.D.) equals to 0.8, 1.5, and 1.2 for *C. tuberculostearicum*, *C. jeikeium*, and *S. epidermidis*, respectively at 600 nm as illustrated by the dotted arrows. *B*. Bacteria were subjected to serial dilutions and plating on Tryptic Soy agar plates (*C. tuberculostearicum* and *C. jeikeium*) or No. 3 Agar plates (*S. epidermidis*). CFU/mL were determined at dilutions of 10^-6^, 10^-5^, and 10^-4^. The average bacterial counts were calculated as 8.8 x 10^7^ CFU/mL (*C. Jeikeium*), 1.2 x 10^7^ CFU/mL (*C. tuberculostearicum*), and 1 x 10^8^ (*S. epidermidis*). These numbers were then used to determine the multiplicity of infection (MOI) required to infect the cultured skin cells (HEKs or SCC), Data, N = 4, error bars ± SD.

**Table S1. Primers used for PCR analysis**

Forward (F) and reverse (R) primer sequences (5′- 3′) are shown in addition to the accession number for each gene. All primers were designed using Primer Express software (Applied Biosystems) and were synthesized by the DNA synthesis lab at the University of Calgary.

| **Gene name** | **Accession Number** | **Q-PCR primer sequence (5**′**→3**′**)** |
| --- | --- | --- |
| RPL19 | NM_000981.4 | **F:** ATCGATCGCCACATGTATCA  **R:** GCGTGCTTCCTTGGTCTTAG |
| EGF | NM_001178130.2 | **F:** ACACATGCTAGTGGCTGAAA  **R:** GCATCCTCTCCCTCTGAAATAC |
| IL1ra | NM_000577.4 | **F:** AGCTGCCTGCAGTACTTTAC  **R:** CCTTCAGCAGAGGAAGAAAGAA |
| IL2 | NM_000586.3 | **F:** CTCACCAGGATGCTCACATTTA  **R:** CCTCCAGAGGTTTGAGTTCTTC |
| IL3 | NM_000588.3 | **F:** CTGGGTCATCTCTCACACATTC  **R:** ATGGGAGCTGCACATTTCA |
| IL4 | NM_000589.4 | **F:** GTTCTACAGCCACCATGAGAA  **R:** CCGTTTCAGGAATCGGATCA |
| IL5 | NM_000879.3 | **F:** GGATGCTTCTGCATTTGAGTTT  **R:** CAGTGCCAAGGTCTCTTTCA |
| IL6 | NM_000600.5 | **F:** TGACCCAACCACAAATGC  **R:** AGGAACTCCTTAAAGCTGCG |
| IL7 | NM_000880.4 | **F:** CTTCTTCTGTGCTGGAGATGTT  **R:** GACCTTGTTATGCTGTTGCTTAC |
| IL8 | NM_000584.4 | **F:** CTTGGCAGCCTTCCTGATTT  **R:** GGGTGGAAAGGTTTGGAGTATG |
| IL9 | NM_000590.1 | **F:** GATCCTGGACATCAACTTCCTC  **R:** ACAGAGACAACTGGTCACATTAG |
| IL10 | NM_000572.3 | **F:** TTTCCCTGACCTCCCTCTAA  **R:** CGAGACACTGGAAGGTGAATTA |
| IL13 | NM_001354991.1 | **F:** GCTCTCAGCCAACGAGTAAT  **R:** CAGTGTTCAAGGTACCCTTCTAT |
| IL17a | NM_002190.3 | **F:** CAAGACTGAACACCGACTAAGG  **R:** CCATTCCTCAGGGCCATTATC |
| IL1β | NM_000576.2 | **F:** CAAAGGCGGCCAGGATATAA  **R:** CTAGGGATTGAGTCCACATTCAG |
| IL1α | NM_000575.4 | **F:** CATCCTCCACAATAGCAGACAG  **R:** GAGTTTCCTGGCTATGGGATAAG |
| COX2 | NM_000963.4 | **F:** TACTGGAAGCCAAGCACTTT  **R:** GGACAGCCCTTCACGTTATT |
| CXCL1 | NM_001511.3 | **F:** CCTGCCCTTATAGGAACAGAAG  **R:** AAGCGATGCTCAAACACATTAG |
| CXCL10 | NM_001565.4 | **F:** GTAATAACTCTACCCTGGCACTATAA  **R:** GATGGGAAAGGTGAGGGAAATA |
| ICAM1 | NM_000201.3 | **F:** GTAGCAGCCGCAGTCATAAT  **R:** GGGCCTGTTGTAGTCTGTATTT |
| HBEGF | NM_001945.3 | **F:** AGCTCTTTCTGGCTGCAGTTCTCT  **R:** TCCAGATCTGCCTCTTGCAAGTCA |

**Table S1. Continued**

| IRF1 | NM_001354924.1 | **F:** GTGTGGATCTTGCCACATTTC  **R:** CCGAGCAAGGCACTGTATAA |
| --- | --- | --- |
| IL32 | NM_001012631.1 | **F:** GAGCTCTTCATGTCCTCTTTCC  **R:** GGCAAAGGTGGTGTCAGTAT |
| GMCSF | NM_000758.4 | **F:** GAGCTAGAAACTCAGGATGGTC  **R:** TCTTCTGCCATGCCTGTATC |
| STAT1 | NM_007315.3 | **F:** CACCTACGAACATGACCCTATC  **R:** TAGGACTGGACTGGACTTAGAC |
| TNF | NM_000594.3 | **F:** AGAGGGAGAGAAGCAACTACA  **R:** GGGTCAGTATGTGAGAGGAAGA |
| TGFβ | NM_000660.6 | **F:** CCTGCCTGTCTGCACTATTC  **R:** TGCCCAAGGTGCTCAATAAA |
| CSF3 | NM_000759.3 | **F:** TGTGTCCTTCCCTGCATTT  **R:** TTACCTATCTACCTCCCAGTCC |
| LTB | NM_002341.1 | **F:** CCGACGAGACAGTAGAGGTAATA  **R:** AGGGTGTACGTCAACATCAG |

**Table S2. Primers used for ChIP PCR analysis of NF-κB-P_65_** **and RNA Pol II occupancy at the promoter region of IL1β, IL6, and CSF3 genes**

Forward (F) and reverse (R), Sequence location relative to the transcription start site (TSS) and primer sequences (5′- 3′) are shown. All primers were designed using Primer Express software (Applied Biosystems) and were synthesized by the DNA synthesis lab at the University of Calgary.

| **Gene name** | **Sequence location relative to the TSS** | **ChIP primers sequence (5**′**→3**′**)** |
| --- | --- | --- |
| IL1β | -346 ̶ -468 | **F:** TACAGACAGGGAGGGCTATT  **R:** GTGGGACAAAGTGGAAGACA |
| IL6 | -51 ̶ +50 | **F:** CCTCACCCTCCAACAAAGATT  **R:** CCTCAGACATCTCCAGTCCTAT |
| CSF3 | -218 ̶ -101 | **F:** GCCTTTGTTCAGCTGTTCTG  **R:** TGGAATCTCTGATCCTCCTTTG |

**Table S3. ChIP PCR primers of negative control regions**

Forward (F) and reverse (R) primer sequences (5′-3′) are shown in addition to the accession number for each negative control gene. All primers were designed using Primer Express software (Applied Biosystems) and were synthesized by the DNA synthesis lab at the University of Calgary.

| **Gene name** | **Accession number** | **Sequence (5`→3`)** |
| --- | --- | --- |
| MYOD1 | NC_000011.10 | **F:** TGCAGGAGATGAAATACTAAGCAAGTA  **R:** AGATTGGAAACTGAGGACTTTAGTTAGAG |
| OLIG3 | NC_000006.12 | **F:** GGCAAGGACAGAGACAATCATA  **R:** CTCTGTGTTCTCGCTTTGGA |
